# The Genetic Architecture of Oral Language, Reading Fluency, and Reading Comprehension: A Twin Study From 7 to 16 Years

**DOI:** 10.1037/dev0000297

**Published:** 2017-06

**Authors:** Maria G. Tosto, Marianna E. Hayiou-Thomas, Nicole Harlaar, Elizabeth Prom-Wormley, Philip S. Dale, Robert Plomin

**Affiliations:** 1 Department of Psychology and Laboratory for Cognitive Investigations and Behavioural Genetics, Tomsk State University; 2 Department of Psychology, University of York; 3 Department of Pediatrics, University of Colorado School of Medicine, Anschutz; 4 Division of Epidemiology, Virginia Commonwealth University; 5 Department of Speech and Hearing Sciences, University of New Mexico; 6 Social, Genetic, and Developmental Psychiatry Research Centre, Institute of Psychiatry, King’s College London

**Keywords:** genetic architecture, twin study, language, reading fluency, reading comprehension

## Abstract

This study examines the genetic and environmental etiology underlying the development of oral language and reading skills, and the relationship between them, over a long period of developmental time spanning middle childhood and adolescence. It focuses particularly on the differential relationship between language and two different aspects of reading: reading fluency and reading comprehension. Structural equation models were applied to language and reading data at 7, 12, and 16 years from the large-scale TEDS twin study. A series of multivariate twin models show a clear patterning of oral language with reading comprehension, as distinct from reading fluency: significant but moderate genetic overlap between oral language and reading fluency (genetic correlation r_g_ = .46–.58 at 7, 12, and 16) contrasts with very substantial genetic overlap between oral language and reading comprehension (r_g_ = .81–.87, at 12 and 16). This pattern is even clearer in a latent factors model, fit to the data aggregated across ages, in which a single factor representing oral language and reading comprehension is correlated with—but distinct from—a second factor representing reading fluency. A distinction between oral language and reading fluency is also apparent in different developmental trajectories: While the heritability of oral language increases over the period from 7 to 12 to 16 years (from h^2^ = .27 to .47 to .55), the heritability of reading fluency is high and largely stable over the same period of time (h^2^ = .73 to .71 to .64).

There is a long-established and robust relationship between oral language skills and literacy, which has been explored extensively using behavioral methodologies, and more recently with neuroimaging and genetic techniques. Language skills are highly predictive of progress in reading, both in the early acquisition of decoding, and in later stages when the emphasis shifts to comprehension of the text (Chall, 1983). The widely recognized Simple View of Reading (Hoover & Gough, 1990) suggests that reading comprehension skills can be conceptualized as the product of decoding skills and oral language comprehension. According to this theory, oral language comprehension and reading comprehension are very closely aligned, but their relationship strengthens as decoding becomes well established and no longer constrains fluent reading.

Most of the research on this relationship has been carried out with children in early and middle childhood, when reading is explicitly taught as a skill, and when there are substantial individual differences in the ease and speed with which children learn to read. Considerably less is known about these processes in adolescence, but it is important to examine them in this period as well, for several reasons: First, research over the last 10 years has demonstrated that the adolescent brain continues to develop substantially into the late teens and early twenties, so that a full characterization of learning processes needs to take this period into account (Blakemore, 2012). Second, as reading skills—especially decoding—become established in the later primary school years, a strong implication of the Simple View is that reading comprehension and oral language skills become highly overlapping. Recent neuroimaging evidence supports the claim that by late adolescence the neural systems underpinning reading and spoken comprehension have converged to form an “abstract supramodal language system” (Braze et al., 2011). It is important to establish the time-course of this emergent system and the biological factors that shape it, including the fundamental influences of genetics and environments.

Third, and relatedly, there continue to be wide variations in both oral language and literacy skills throughout secondary school and adult life. Since literacy is an important key to academic and occupational success, children who fail to make the transition successfully from “learning to read” to “reading to learn” are likely to be at a particular disadvantage.

The present study focuses on the sources of variation in oral language and literacy skills over a wide developmental window, from the age of 7, when children are still learning to read, through to age 16, when many children attain adult levels of reading (van den Bos, Zijlstra, & Lutje Spelberg, 2002). We utilize a twin design to identify the relative contributions of genetic and environmental factors to individual differences in oral language, reading fluency, and reading comprehension skills at the ages of 7, 12, and 16. Multivariate genetic analyses then allow us to examine our primary questions, which are concerned with the changing relationships between aspects of reading and language with development.

## Genetic and Environmental Influences on Reading Development

Previous work using behavioral genetic methodology has demonstrated the importance of genetic factors in reading and related skills. The basic findings have been replicated in diverse twin samples in the U.S., United Kingdom, Australia. Scandinavia, and China, which have shown high heritability (h^2^ = ∼.70) for word and nonword reading on tests of early decoding and reading efficiency (Chow et al., 2011; Harlaar, Spinath, Dale, & Plomin, 2005; Taylor & Schatschneider, 2010; Samuelsson et al., 2008). Genetic influences on word-level reading are consistently high when measured during or after the first year of formal reading instruction, and remain at similar levels from the end of kindergarten through to fourth grade (Byrne et al., 2009; Christopher et al., 2013a; Petrill et al., 2007). An interesting exception to this otherwise remarkably consistent picture is when reading skills were measured at the end of kindergarten in Scandinavia, where shared environmental influences were dominant (52%) and genetic influences weaker (33%; Samuelsson et al., 2008). The likely explanation for this is that formal reading instruction begins in first grade in Scandinavia—a year later than in the other educational systems in these studies—and that prior to this, variation in the home and preschool environments exerts a strong effect on early literacy skills; once formal schooling begins, it substantially reduces the environmental variance.

Genetic effects also appear to play an important role in reading comprehension. Moderate to high genetic and low shared environmental effects have been reported for a variety of different measures of reading comprehension in both middle childhood and adolescence, with heritability estimates usually in the region of 50%–60% (Byrne et al., 2009; Harlaar et al., 2010; Harlaar, Dale, & Plomin, 2007; Olson et al., 2011). Although different measures of reading comprehension vary in terms of the extent to which they draw on word-level reading versus higher-level comprehension skills, this does not appear to affect the level of heritability (Betjemann et al., 2011).

Longitudinal twin studies have also been used to go beyond estimating genetic and environmental effects on reading at individual time points to address the role of genetic and environmental influences on reading development across time. Behavioral studies focusing on longitudinal development consider the overall levels of observed stability in reading over time; behavioral genetic studies can extend this by considering the extent to which the observed levels of stability or change are due to genetic or environmental influences that continue to influence reading over the course of development. The evidence to date suggests high levels of genetic and environmental stability for both word-level and reading comprehension skills across the primary school years (Harlaar et al., 2007; Logan et al., 2013; Olson et al., 2011), as well as into adolescence (Hulslander et al., 2010). These results suggest that the underlying genetic factors that influence children’s early reading skills continue to exert their effects later on, and that most of the observed phenotypic stability in reading can be accounted for by genetic factors. Importantly, this stability appears to be present not only in the early years when children are learning to read, but also across the transition to “reading to learn” which occurs in the later primary school years (Harlaar, Dale, & Plomin, 2007).

Most recently, biometric growth models have been applied to these data to try to tease apart the etiology of the intercept—children’s starting level of reading—and that of the subsequent rate of growth. The results regarding the intercept are generally consistent with the previous literature in showing large genetic influence on variation in the starting level of reading. Further, genetic variance is also important for the subsequent rate of growth in early reading skills (Christopher et al., 2013b; Logan et al., 2013).

## Genetic and Environmental Influences on Oral Language Development

A smaller body of work has examined the etiology of individual differences in oral language skills. Genetic influences appear to be significant from the emergence of spoken language in the toddler years onward, but there are also substantial shared environmental effects that are important drivers of early language skills; these are actually larger than the genetic effects, at least in the preschool years (Chow et al., 2011; Hayiou-Thomas et al., 2006; Olson et al., 2011; Spinath et al., 2004). This pattern changes as children grow older, such that genetic influences become stronger from middle childhood onward, and shared environmental effects become weaker: in the International Longitudinal Twin Study (ILTS), the heritability of vocabulary measures increased from 29% in prekindergarten, to 57% in fourth grade (Olson et al., 2011). Similarly, in the United Kingdom-based Twins Early Development Study (TEDS), heritability estimates for latent factors of oral language increase from approximately 30% in 2-, 3-, and 4-year-old children, to 60% in 12-year-olds, while estimates of the shared environment decrease from 60% to 20% (Hayiou-Thomas et al., 2012). While it may seem counterintuitive that genetic influences become more dominant with development—as individuals accumulate experience—this pattern has been well-documented in other domains, most notably “g” (Haworth et al., 2010). In terms of stability over time, the TEDS data suggests a pattern of lower stability—both phenotypic and genetic—between early and middle childhood, with high levels of stability thereafter.

## Genetic and Environmental Overlap Between Oral Language and Reading

Genetic and environmental factors influencing individual variations in preschool speech and language abilities also exert their influence on early literacy. Drawing on data from the TEDS sample, Harlaar, Hayiou-Thomas, Dale, and Plomin (2008) showed that a parent-reported vocabulary and grammar composite in 2-, 3-, and 4-year-olds was moderately predictive of teacher-rated reading achievement in the primary school years (ages 7, 9, and 10). This relationship was primarily mediated by a common set of shared environmental influences, which played a large role in early language skills, and a relatively small role in later reading; there was also a smaller effect of genetic factors that influenced both early language and later reading. A further analysis focused specifically on the contrast between broad oral language skills (including vocabulary, grammar, semantic fluency, and narrative recall), and speech skills in a subset of TEDS twins assessed at 4 1/2 years of age. As before, common environmental as well as genetic influences contributed to the relationship between broad oral language skills and later reading, but only genetic factors contributed to the relationship between speech production skills and reading (Hayiou-Thomas et al., 2010).

A particularly close relationship has been documented between reading comprehension and oral language skills. The Simple View of Reading (Hoover & Gough, 1990) also appears to hold at the genetic level. Using data from the Colorado Learning Disabilities Research Center (CLDRC), Keenan, Betjemann, Wadsworth, DeFries, and Olson (2006) modeled the genetic relationship between decoding skills, reading comprehension, and listening comprehension. They found that two latent genetic factors could account for the pattern of covariance: one that exerted influence on all three measures, and a second factor that influenced listening and reading comprehension, but not decoding. Crucially, there was no specific genetic influence on reading comprehension alone: *all* the genetic (and also shared environmental) variance on reading comprehension was shared with decoding and listening comprehension. This first study of its type reported preliminary analyses based on a relatively small sample of twins, and covering a broad age-range (8–17). However, the findings proved to be robust, as they were replicated in independent samples of 9- to 10-year-old twins participating in the Western Reserve Reading Project (Harlaar et al., 2010), and the International Longitudinal Twin Study (Olson et al., 2011). Interestingly, ILTS data from somewhat younger children (age 7), shows a closer genetic association between reading comprehension and decoding than is found at later ages, presumably because at this early point in learning to read, comprehension is largely constrained by decoding skill (Byrne et al., 2006; Olson et al., 2011).

The existing studies clearly show that reading comprehension shares genetic resources with both decoding and listening comprehension, and also suggest that the pattern of associations may change with age, particularly within the primary school years. This study extends prior work by examining the phenotypic and etiological relationship between reading fluency, oral language skills, and reading comprehension, across the transition into adolescence. We do this by modeling (a) the longitudinal age-to-age continuity *within* each of the three constructs in order to shed light on the relative etiological stability of decoding, oral language and reading comprehension, and (b) the multivariate relationships among these three constructs. In order to contextualize our findings within a broad developmental picture, we also include data on oral language and reading fluency at the age of 7.

The measures and constructs we focus on are very similar to those in previous studies, but not identical. First, we focus on measures of reading fluency, rather than word-reading accuracy, because in adolescence the majority of children are accurate readers of single words, but there is still substantial variability in the fluency with which they read. Second, rather than using a single measure of listening comprehension, the aspect of oral language that the Simple View of Reading focuses on, we assessed a diverse range of skills, including vocabulary, grammar, figurative language, and inference-making. Our measures of reading comprehension include tests of both sentence- and passage-level comprehension, and are similar to those used in previous studies.

Based on previous work in the field, our hypotheses with respect to the levels of heritability at different ages for language and reading skills are as follows:

1 The heritability of oral language skills will be moderate, and will increase with age.
2 The heritability of reading fluency will be high, and its magnitude will not increase over time.
3 The heritability of reading comprehension will be moderate to high, but no specific prediction is made with respect to changing levels of heritability with age.

With respect to the multivariate relationships between oral language, reading fluency, and reading comprehension, we hypothesize that:

4 There will be significant genetic and environmental overlap between all three constructs, but a closer association between oral language and reading comprehension, than between oral language and reading fluency.
5 The strength of this association will change with age: we predict that there will be a greater differentiation between oral language and reading fluency in adolescence (ages 12 and 16), than in middle childhood (age 7). We do not make a specific prediction with respect to age and the relationship between reading comprehension and either oral language, or reading fluency.

## Method

### Participants

The sampling frame for the present study is the United Kingdom-based Twins Early Development Study (TEDS), an ongoing longitudinal twin study (Haworth, Davis, & Plomin, 2013). After checking for infant mortality, all families identified by the United Kingdom Office for National Statistics (ONS) as having twins born between 1994 and 1996 were invited to participate in TEDS when the twins were about 18-months-old. The twins have been assessed on measures of language, cognitive, and behavioral development at regular intervals from the age of 2 onward, using a variety of methods, including parent questionnaires, telephone testing, and web-based assessment. The current study focuses on data collected at the ages of 7, 12, and 16.

Twin pairs were excluded where either member of the pair had any major medical or perinatal problems, documented hearing loss, or organic brain damage. Zygosity was determined in same-sex twin pairs by a well-validated parental questionnaire completed at 2, 3, and 4 years (Price et al., 2000), with follow-up testing of polymorphic DNA markers in uncertain cases. In all selected families for the current study, English was the only language spoken at home. The current study is based on the resulting sample of twin pairs, with data at each of the following ages: age 7, *N* = 7,319 pairs, mean age 7.16 (.26); age 12, *N* = 6,858 pairs, mean age 11.72 (.65); age 16, *N* = 6,689 pairs with mean age 16.48 (.27). The specific sample size for each measure and analysis is reported in Tables 1, 2, and 3.[Table-anchor tbl1][Table-anchor tbl2][Table-anchor tbl3]

The TEDS sample has continued to be reasonably representative of the United Kingdom population with respect to ethnicity, maternal education and employment, and paternal employment (Haworth et al., 2013).

### Measures

#### Oral language

##### 7 years

At age 7, children’s oral language skill was indexed by expressive vocabulary, administered over the telephone, using the vocabulary subtest of the WISC–III (Wechsler, 1992; split-half *r* = .79; test–retest *r* = .82)

##### 12 years

Participants were assessed on a web-based battery of the following four receptive language measures. As we have previously shown that these measures are closely related etiologically (Dale et al., 2010), we created a composite (averaging their standardized means) for the purposes of the current analyses.

#### Vocabulary

The WISC–III-PI Vocabulary Multiple Choice subtest was used (Kaplan et al., 1999; split-half *r* = .93; test-retest *r* = .58).

#### Nonliteral semantics

In addition to vocabulary, semantics was assessed using the Figurative Language subtest of the Test of Language Competence—Expanded Edition, Level 2 (Wiig et al., 1989; α = .67; test–retest *r* = .73). This subtest assesses the interpretation of idioms and metaphors.

#### Syntax

Syntax was assessed using the Listening Grammar subtest of the Test of Adolescent and Adult Language (TOAL-3; Hammill et al., 1994; α = .94; test–retest *r* = .81). Children were required to select two sentences that have nearly the same meaning from a set of three options.

#### Pragmatics

The Making Inferences subtest of the Test of Language Competence requires participants to make permissible inferences on the basis of existing, but incomplete, causal relationships in the context of short paragraphs presented orally. (Wiig et al., 1989; α = .71; test–retest *r* = .54).

#### 16 years

Similar to age 12, two web-based tests were used to assess language at age 16, and a composite of these was used in the current analyses (*r* = .48).

#### Vocabulary

Vocabulary was tested with the Mill Hill Vocabulary test, Set B (Raven, Court, & Raven, 1998). The participant saw a single word presented at the top of the screen, and had to choose the word closest in meaning from a list of six options listed below. The initial 11 items were dropped in this web-based version of the test, as they had previously been found not to contribute any variance. (α = .81; test–retest *r* = .64).

#### Nonliteral semantics

The Figurative Language subtest of the Test of Language Competence was used as at 12, with an additional four items to extend the range at the upper end. (α = .69; test–retest *r* = .71).

#### Reading efficiency

##### 7 years

The Test of Word Reading Efficiency, Form B (Torgesen, Wagner, & Rashotte, 1999) was included in a test booklet sent to families by mail (one test booklet for each twin), and was administered to each twin separately by telephone. In the Sight Word Efficiency subtest, children were given 45 seconds to read aloud as many words as they could from a list in front of them. In the Phonemic Decoding Efficiency subtest, the list was comprised of nonwords. As previous analyses have shown that the two subtests are highly intercorrelated (*r* = .83, Dale, Harlaar, & Plomin, 2005), a composite of the two subtests was used in the current analyses. Alternate forms (Form B at 7 years and Form A at 9 years) correlation, *r* = .83; this can be seen as a lower-limit estimate of reliability.

##### 12 years

As at 7, the TOWRE was administered to children over the telephone. In addition, children completed an online adaptation of the Woodcock-Johnson III Reading Fluency test (W-J III; Woodcock et al., 2001). In this timed test, children had to respond yes or no to a series of simple sentences (“Ants are very big”); the total number of correct responses within 3 min was summed to give a total fluency score (α = .96; test–retest *r* = .81). A composite of the TOWRE and W-J III Reading Fluency (average of their standardized means) test was used in the analyses (*r* = .56).

##### 16 years

The online adaptation of the reading fluency subtest from the Woodcock-Johnson III was used again at 16, with the time limit reduced to 2.5 min.

#### Reading comprehension

##### 12 years

Sentence-level reading comprehension was assessed using a web-based version of the Reading Comprehension subtest of the Peabody Individual Achievement Test (PIAT; Markwardt et al., 1997), in which children read a sentence and chose the matching picture from a set of four. (α = .94; test–retest *r* = .80). In addition, children completed a web version of the GOAL Formative Assessment in Literacy for Key Stage III (GOAL plc, 2002), which includes a wide range of literal and inferential comprehension questions. Children read the stimulus sentence or short paragraph, and selected the appropriate answer for a set of four multiple choice options. (α = .91; test–retest *r* = .52). A composite of these two measures of reading comprehension (average of their standardized means) was used in the current analyses (*r* = .58).

##### 16 years

Our reading comprehension measure was modeled on the York Assessment of Reading Comprehension (YARC; Snowling et al., 2009). Several passages that had been created for the YARC but not used were generously shared by the developers and evaluated in pilot work. Two passages, one fiction and one nonfiction, were selected, and the 13 questions for each of those passages were converted from an open-ended response format to multiple choice format suitable for web administration. (α = .72; test–retest *r* = .63).

### Genetic Analysis

Genetic analyses were based on the twin design, which capitalizes on the fact that identical (MZ for monozygotic) twins share 100% of their varying DNA while fraternal twins (DZ for dizygotic) share on average 50% (Plomin, DeFries, Knopik, & Neiderhiser, 2013). Overall similarity of individuals within a twin pair, regardless of zygosity, indicates familiality; however, if the members of an MZ twin pair are more similar to each other on a given trait than the members of a DZ pair, it can be inferred that genetic factors play a role in driving individual differences in that trait. Comparing the MZ and DZ twin similarity (similarity computed as correlation within each twin pair: Intraclass Correlation [ICC]) on a single trait yields an estimate of univariate heritability. Heritability indexes the extent to which individual differences on the trait are caused by genetic as opposed to environmental factors. It is possible to extend this model to examine the origins of the covariance between two or more measures by comparing Trait 1 in Twin 1 to Trait 2 in Twin 2 (Martin & Eaves, 1977), and that multivariate approach is at the core of the present analyses.

The current analyses were based on raw data, and used the structural equation modeling package OpenMx (Boker et al., 2012, 2011). The basic genetic model employed uses the maximum likelihood method to obtain parameter estimates for the effects of additive genetic (A), shared environmental (C), and nonshared environmental (E) influences on a given trait. The additive genetic and shared environmental influences are what make the children within a twin pair similar to each other, while the nonshared—or unique—environmental influences contribute to differences within the pair. The E parameter also includes the effects of measurement error. The model assumes that there are no effects of nonadditive genetics, nonrandom mating, or gene-environment interaction. The genetic analysis used scores that were corrected for the linear effects of age and sex, as these can inflate twin similarity (McGue & Bouchard, 1984).

Prior to the main analyses of interest, we carried out sex-limitation analyses for each construct at each age, to ascertain whether there were qualitative sex-differences (different genetic factors influencing behavior in the two sexes), quantitative sex-differences (the same genetic factors in the two sexes, but affecting one sex more than the other), or variance differences (no genetic differences, but different phenotypic variance in the two sexes). In most cases, the null model was the best-fitting model, indicating no sex differences. The two exceptions were (a) the reading comprehension composite at age 12, for which the model parameters suggested a marginally significant, but very small, quantitative sex-difference; and (b) reading fluency at age 7, for which there were small significant differences between the sexes in phenotypic variance, but no evidence of genetic differences. Taken together, the sex-limitation analyses do not provide evidence of genetic sex-differences in our language or reading measures. However, they do show significantly greater phenotypic variability for reading fluency in 7-year-old boys compared with girls. Full details of the model-fitting parameters for the sex-limitation models are available as online supplementary material. Given the lack of sex-differences, DZ opposite sex twins were included in all genetic models presented, thus maximizing statistical power.

To examine the magnitude of genetic and environmental effects over time for each of our three constructs, we used Cholesky decomposition models, which estimate the relative contributions of A, C, and E sources of variance to the measures at each age. The model allows for a new A, C, and E factors at each age for each variable, so that it is possible to examine whether genetic (and environmental) influences at age 7 also contribute to variance in the measures at ages 12 and 16; and whether there are additional genetic influences that are specific to ages 12 and 16.

The multivariate relationships between language and reading measures were modeled separately for each age, in the first instance, using correlated factors models. These yield estimates of the degree of overlap in the etiology of language and reading: the genetic correlation (r_g_) provides an estimate of the extent to which it is the same or different genes which affect the measures, independent of their heritabilities. Similarly, the shared environment correlation (r_c_ and r_e_ respectively) and the unique environmental correlation estimate the extent to which same or different environmental factors are influential.

Finally, we used a common pathways genetic model in order to examine the etiological relationship between oral language, reading efficiency, and reading comprehension, irrespective of age. In this model the measured variables from ages 7, 12, and 16 are hypothesized to load onto two latent factors, representing (a) reading efficiency, and (b) comprehension. The model provides estimates for these factor loadings. The etiology of the latent factors is then partitioned into the proportions of their variance explained by additive genetic (A), shared environment (C), and nonshared environment (E). The degree of overlap between the latent factors is reflected in the genetic and environmental correlations, and the model also estimates A, C, and E parameters for influences that are specific to each of the measured variables. Importantly, the specific E parameters also incorporate measurement error. Finally, there are estimates for the total effects of A, C, and E on each of the measures, which combine the shared and measure-specific effects.

## Results

### Phenotypic Analyses

Means and standard deviations for the measures, divided by sex and zygosity, are presented in Table 1. The table also presents a summary of the ANOVA testing the effects of sex and zygosity on the measures. Due the large sample size, small significant effects of sex and zygosity were detected; however these explained very little of the variance in all variables (*R*^2^ between 0% and .4%). The effects of sex were significant for reading fluency at all three ages, favoring girls, but not for language or reading comprehension. The effects of zygosity were significant for all measures except for reading comprehension at age 16. There was no interaction of sex and zygosity in any measure.

The phenotypic correlations, presented in Table 2, show moderate to substantial associations between reading and language, both concurrently and longitudinally. In terms of age-to-age stability within constructs, the Language and Reading Comprehension composites had average correlations of *r* = .48 and .49, respectively, while the average correlation for reading fluency measures across ages was somewhat higher, at *r* = .63. Across constructs, the correlations between language and reading comprehension were high, averaging *r* = .60, while the average correlations between reading fluency and language, and between reading fluency and reading comprehension were both lower, at *r* = .41. The pattern of phenotypic correlations, both longitudinally within constructs, and concurrently across constructs, suggest that language and reading comprehension pattern together, with reading fluency slightly separate.

### Univariate Genetic Analyses

Intraclass correlations indexing the twins’ similarity on reading and language, are presented in Table 3. For all measures, MZ twin correlations were greater than DZ twin correlations, suggesting genetic influences on individual differences in reading and language skills across the three ages. Heritability estimates derived from univariate model fitting analyses are presented in Table 3 for all measures (model-fitting statistics for this set of models are presented in the supplementary online material).

Reading fluency showed the highest heritability over time, with an average heritability (h^2^) across the three ages of .69, while reading comprehension and language showed very similar, moderate, heritability over time: .48 and .43, respectively. The effects of shared environment were generally small and decreased with age; the largest effects were observed for language at age 7 (.37), while zero value for this estimate was observed for reading comprehension at age 16. Nonshared environmental effects were modest and significant for all measures at all ages; note, however, that these estimates include measurement error.

### Longitudinal Genetic Analyses of Language and Reading

**Figure 1 fig1:**
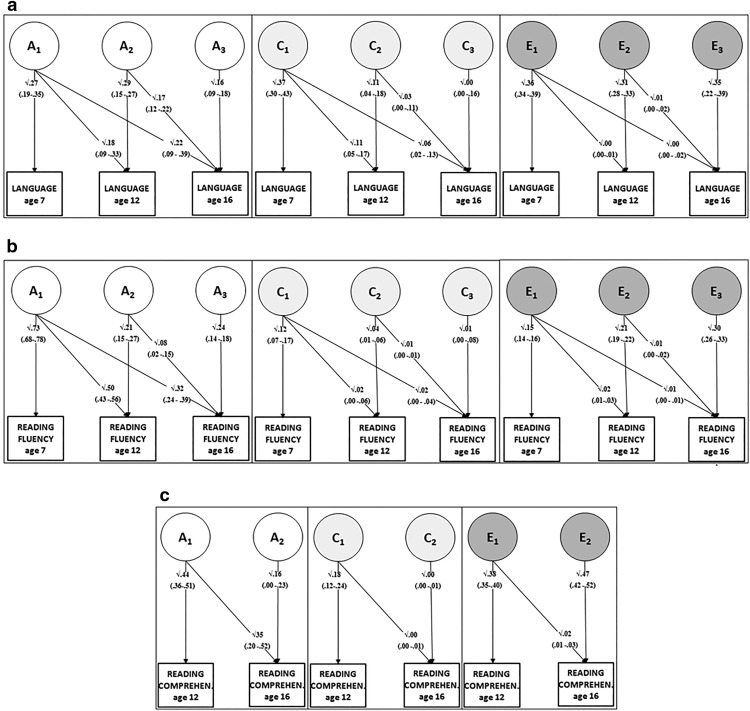
(a) Longitudinal Cholesky model for oral language, ages 7, 12, and 16. The figure summarizes genetic and environmental influences on each measure at a specific time and in common over time. The straight paths from each latent variable to the measure represent the genetic (paths from As), shared environmental (paths from Cs) and nonshared environmental (paths from Es) influences specific at each time. The oblique paths represent time-shared genetic and environmental influences. For example, unique genetic influences on language at age 7 are represented by the straight path from A_1_ with coefficient √.27 (this also represents heritability of language at age 7). The time specific influences at age 12 and 16 are represented by the vertical paths from A_2_ (√.29) and from A_3_ (√.16), respectively. The diagonal path from A_1_ with coefficient √.18 represents genetic factors influencing language both at age 7 and 12, the path coefficient √.22 represents genetic influence in common between age 7 and 16 but not with age 12, while the diagonal path from A_2_ (√.17) shows the genetic influences common between ages 12 and 16 but not 7. The same logic applies to the shared and nonshared environmental influences. (b) Longitudinal Cholesky model for reading fluency, ages 7, 12 and 16. (c) Longitudinal Cholesky model for reading comprehension, ages 12 and 16.

We examined the genetic architecture of stability and change over time, for each of the three constructs of language, reading comprehension and reading fluency (Figure 1; model-fitting statistics are presented in Table 3 in the supplementary online material).[Fig-anchor fig1]

#### Language

It is apparent from the estimates in Table 3 that the heritability of language increases with age, particularly from ages 7 to 12. The Cholesky decomposition presented in Figure 1a suggests that this increase in heritability is partly due to the continuation of early genetic influences at later time points, which is shown by the significant path coefficients on the diagonal lines from earlier genetic latent factors to later language measures (A_1_ to age 12 and 16 language; A_2_ to age 16 language). In addition, there is evidence of genetic innovation—new genetic influences contributing to the increase in heritability—at both ages 12 and 16; this is reflected in the significant path coefficients from A_2_ and A_3_ to age 12 and 16 language measures. It is worth noting that the size of novel genetic effects unique to age 16 (A_3_) is small relative to the contribution of earlier genetic effects (A_1_ and A_2_). The genetic correlations (see Table 4) further clarify the pattern, by showing substantial—but not perfect—continuity in terms of genetic factors that influence language from age 7 to age 12 (r_g_ = .63), and an even higher level of continuity/stability from age 12 to age 16 (r_g_ = .89). To a much lesser extent than the genetic factors, shared environment also contributes to longitudinal stability in language skill: while the overall effect of shared environment diminishes over time, it appears to be the same factors that were present at 7 years (C_1_) that continue to account for most of the (modest) shared environmental influence at the later ages, with a small additional age-specific effect at age 12. The effects of nonshared environment, by contrast, are unique to each age, and do not contribute to longitudinal stability (note that this variance may also represent method variance or measurement error). In summary, the steep increase in heritability for the language measures from ages 7 through to 16 seems to be due to a combination of early genetic influences which continue to exert their effects over time, and novel genetic effects that come into play in adolescence.[Table-anchor tbl4]

#### Reading fluency

A rather different pattern emerges for reading fluency, which shows high levels of heritability from age 7 onward: the overlapping confidence intervals indicate that there is no change in the magnitude of the heritability estimates at ages 7, 12, and 16. As the Cholesky decomposition (Figure 1b) illustrates, there is evidence of some genetic innovation, in the form of significant contributions from latent genetic factors A_2_ and A_3_ at ages 12 and 16. However, the magnitude of the novel effects is relatively small compared with the genetic effects that are carried forward from age 7 (A_1_). This suggests that it is largely the same genetic factors already present by age 7, which continue to be the main drivers of individual differences in decoding skills at ages 12 and 16. A high level of genetic stability is further illustrated by the very high age-to-age genetic correlations (see Table 4): age 7–age 12 r_g_ = .84; age 12–age 16 r_g_ = .78, and age 7–age 16 r_g_ = .72. In contrast to the genetic effects, there is little evidence that environmental effects contribute to stability over time for reading fluency: Shared environmental effects are extremely small, and not significantly different from zero after age 7, while the modest nonshared environmental effects are unique for each age. Overall, genetic factors play a substantial role in reading fluency from the early stages of learning to read at age 7, through adolescence to age 16, and it seems to be largely the same genetic factors influencing reading across this wide age-range.

#### Reading comprehension

Reading comprehension was assessed only at ages 12 and 16, and it is clear that there is substantial genetic stability over this age range. Not only are the heritability estimates very similar at the two ages, but a large proportion of the genetic effects at age 16 are carried over from age 12 (significant diagonal path from A_1_ to age 16 reading), with a much smaller genetic effect that is unique to age 16 reading (A_2_). This stability is also apparent in the very substantial genetic correlation from age-to-age, of r_g_ = .83 (see Table 4). As with reading fluency, environmental factors do not appear to contribute to stability: although there are significant (modest) shared environmental effects at 12, these are nonsignificant at age 16, and the moderate nonshared environmental effects are unique for each age. In summary, although the genetic effects on reading comprehension seem to be smaller for reading comprehension than for reading fluency, reading comprehension remains stable from ages 12 to 16, and this is largely due to stable genetic influences. Details of the multivariate-longitudinal model fitting are presented in the supplementary online material.

### Multivariate Genetic Analyses: The Relationship Between Language, Reading Fluency, and Reading Comprehension

We took two complementary approaches to examining the multivariate relationships between language, decoding and reading comprehension. First, we modeled these relationships at each age separately, in order to examine whether the strength of the genetic and environmental associations across constructs varies with age. Second, we pooled the data across ages to create latent factors with enhanced reliability for language and reading measures, in order to build a robust model of the underlying genetic and environmental architecture irrespective of age.

#### Age-specific models

**Figure 2 fig2:**
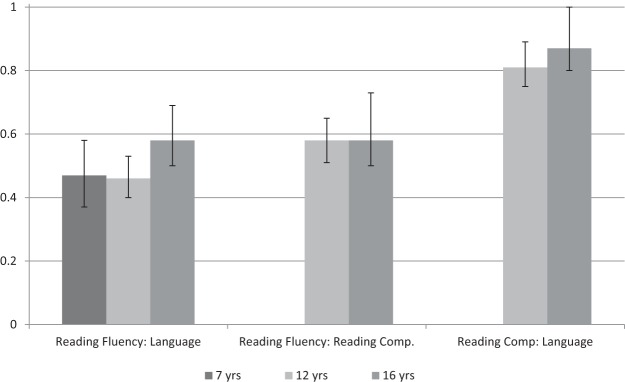
Magnitude of genetic correlations (with 95% CI) at the ages of 7, 12, and 16, between reading fluency and language, reading fluency and reading comprehension, and reading comprehension and language.

We used correlated factors models, focusing on the association between oral language and reading fluency at age 7, and between oral language, reading fluency and reading comprehension at ages 12 and 16. The parameters of interest are the genetic and environmental correlations, which are summarized in Figure 2 and Table 5 (model-fitting statistics are in the supplementary online material in Table 3). The genetic correlation between oral language and reading fluency is moderate and very similar at each age (r_g_ = .47–.58, with overlapping 95% confidence intervals). The genetic correlation between reading comprehension and reading fluency is at a similar level, and identical at ages 12 and 16 (r_g_ = .58). In contrast, the genetic correlation between reading comprehension and oral language at both 12 and 16 is very high (r_g_ = .81–.87, respectively), with the upper confidence intervals approaching unity. The pattern is similar to the phenotypic correlations described earlier, but clearer, in that the association between language and reading comprehension is closer, and the dissociation from reading fluency greater, at the genetic than at the phenotypic level. In contrast to the phenotypic and genetic correlations, the shared environmental correlations shown in Table 5 are consistently high across all three constructs, and the unique environmental correlations consistently low. Strikingly, the multivariate estimates across measures are extremely similar at each of the ages examined, suggesting that underlying etiology of the relationships across language and reading constructs does not change with development.[Fig-anchor fig2][Table-anchor tbl5]

#### Latent factors model, across ages

**Figure 3 fig3:**
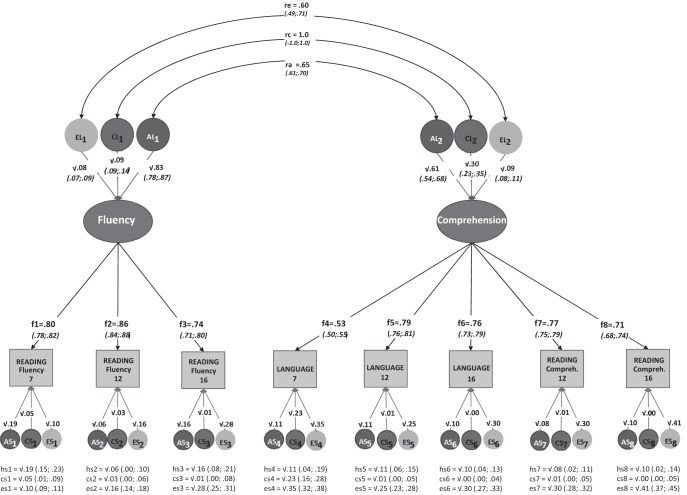
Common pathways model summarizing the genetic and environmental contributions to the relationship between oral language, reading fluency, and reading comprehension across development. r_a_, r_c_, r_e_ = genetic, shared and nonshared environmental correlation between the two latent factors. The paths between each of the genetic latent factors AL, CL, EL and the latent factors of ‘Fluency’ and ‘Comprehension’ represent the genetic, shared and nonshared environmental influences on each of the latent factors. The paths from each variable-specific genetic latent factor, AS, CS, ES, and each variable, represent the variable-specific genetic, shared and nonshared environmental influences. The 95%CI for each of the variable-specific estimates are detailed below each variable by hs, cs and es indices.

We tested a latent factor common pathways model to examine the relationship between language, reading fluency and reading comprehension, aggregated across ages. The focus of this model is on the multivariate relationships among these constructs, rather than on longitudinal stability or change over time. A set of nested models were compared, in which the measured variables loaded onto either one, two or three latent factors (model-fitting statistics in Table 6). The most parsimonious model which fit the data well was a two-factor model^1^ (see Figure 3), in which the reading fluency measures from ages 7, 12, and 16 loaded onto the first factor, while the language and reading comprehension measures loaded onto a second factor.^2^ The factor loadings for all measured variables were generally high, with the weakest loading from 7-year language. The latent factors represent the common variance across measures, and in this case the etiology of the latent factors—presented at the top of Figure 3—can be interpreted as the genetic and environmental effects that are shared across ages (that is, they reflect the longitudinal stability of the constructs). Effects that are specific to any given measure/age are also partialed into genetic and environmental influences, and are presented at the bottom of Figure 3. Note that measurement error in latent factor models is included in the measure-specific e^2^ parameter estimates; the e^2^ estimate for the latent factors, by contrast, is essentially error-free and represents true nonshared environmental variance.[Table-anchor tbl6][Fig-anchor fig3]

The etiology of the latent factors confirmed the high heritability of reading fluency (h^2^ = .83), with only minimal environmental effects (c^2^ = .09, e^2^ = .08). The latent factor for language and reading comprehension, on the other hand, showed moderate effects of shared environment (c^2^ = .30) in addition to the substantial genetic effects (h^2^ = .61). The genetic and environmental correlations for the two latent factors (top of Figure 3) indicated substantial—but not complete—overlap in the genetic influences affecting reading fluency and language/reading comprehension. The shared environmental correlation was 1, suggesting complete overlap in the shared environmental effects on the two factors, although the actual magnitude of these effects on reading fluency is minimal. Similarly, although the nonshared environmental correlation was large, the overall magnitude of these effects was minimal for both factors. Finally, the residual A and C estimates (bottom of Figure 3) show only small age-specific influences for any of the measures (with the possible exception of age-specific C for 7-year language); the age-specific E estimates, which incorporate measurement error, are moderate and significant for all measures. In summary, the multivariate models confirm the pattern observed at individual ages. The robust latent factors approach, in particular, clearly shows that in terms of the underlying etiology, oral language and reading comprehension skills are indistinguishable, and that these are separate from—though related to—reading fluency.

## Discussion

The combined results from our phenotypic and genetic analyses, both longitudinal and multivariate, suggest an underlying etiological divide not between spoken and written language, but between code-based and meaning-based aspects of language and literacy. Although there is a high background level of both phenotypic and etiological association across all three constructs, consistent with the idea of “generalist genes” influencing common aspects of cognition (Kovas & Plomin, 2006), the multivariate latent factors model nonetheless shows that oral language skills and reading comprehension are indistinguishable in terms of their etiology, but that they are both dissociable from reading fluency. Two factors—reading fluency and comprehension—are sufficient to describe the variance, and although they are correlated, they are not the same. Furthermore, this pattern appears to be stable across development from the early stages of learning to read all the way through to mid-adolescence. Although it was important to examine the multivariate relationships separately at each age, the genetic architecture is most clearly captured by the latent factors model that effectively collapses across ages.

The magnitude of genetic and environmental effects also differs for the two factors. While individual differences in reading fluency are driven almost entirely by genetic sources, there are significant shared environmental influences on language/reading comprehension which account for one third of the variance in this factor, alongside the genetic effects. There is also evidence that the developmental trajectories differ in terms of etiology: the longitudinal models showed that reading fluency is highly heritable and genetically stable from at least the age of 7, while oral language may be subject to novel genetic influences after the age of 7. Strikingly, however, by the age of 12, there appears to be a very high degree of stability for all three constructs (language, reading fluency, and reading comprehension), so that there are minimal new genetic effects at age 16, and extremely high genetic correlations from age 12 to age 16.

### Genetic Characterization of Language and Reading Component Skills

Our finding that oral language and reading comprehension pattern together, and can be distinguished from reading fluency, replicates and extends the previous work in the U.S. (Harlaar et al., 2010; Keenan, Betjemann, Wadsworth, DeFries, & Olson, 2006; Olson et al., 2011) which points to an etiological basis for the Simple View of Reading. Furthermore, the genetic dissociation between oral language and code-based aspects of reading is present from the age of 7, and is maintained at the same level throughout childhood and adolescence.

The very close etiological alignment of language and reading comprehension mirrors the neuroimaging results of Braze et al. (2011), and furthermore suggests that intervention effects may generalize between these domains. Consistent with this, a randomized control trial of interventions for children with reading comprehension difficulties found that the oral language arm was the most effective, particularly in the long term follow-up assessment (Clarke et al., 2010).

### Longitudinal Change and Stability in the Etiology of Language and Reading

The current study also confirms—over a longer time-frame and within the same sample—previous findings focusing on word-level reading skills in middle childhood (Byrne et al., 2006; Harlaar et al., 2007; Petrill et al., 2007) and adolescence (Betjeman et al., 2007), that reading fluency is both highly heritable and very genetically stable. This suggests that the etiology of this code-based skill is set at an early point in its development.

With respect to reading comprehension, we found high levels of genetic stability from ages 12 to 16, similar to those reported for the Colorado twin study across a similar age range (Betjeman et al., 2007, which focused on ages 10 and 16). This converging evidence provides strong support for the idea that the genetic resources for reading comprehension are in place by the age of 10–12. However, it is not clear from the existing literature, and we do not have data within the TEDS sample to address the issue of whether or not there is even earlier stability for reading comprehension.

In contrast to both reading fluency and reading comprehension, oral language shows less longitudinal stability. The current results—drawing on TEDS data from ages 7 to 16—are consistent with earlier analyses incorporating a wide range of language measures from the ages of 2 to 12 (Hayiou-Thomas, Dale, & Plomin, 2012), which show that the heritability of oral language skills appears to increase with age. A similar increase in heritability has also been shown in the ILTS study focusing on vocabulary at Grades 2 and 4 (Olson et al., 2011). Taking these three studies together, it appears that influences on early language have a substantial environmental component, which diminishes with age, while genetic effects increase. Moreover, while the increase in heritability initially appears to be at least partly driven by new genetic influences, these stabilize—as reflected in the high genetic correlations across ages—by the later primary school years. A plausible implication of these longitudinal results is that, for younger children’s language where environmental influences are substantial, the existing range of experiences can be harnessed to boost children’s language skills (Byrne, Khlentzos, Olson, & Samuelsson, 2010). There is a rich literature consistent with this view, showing that factors such as the language input provided by caregivers (Hoff, 2006), and the home literacy environment (e.g., Farrant & Zubrick, 2013; Senechal, Pagan, Lever, & Ouellette, 2008), predict language development. We speculate that in older children and adolescents, where environmental influences on language appear to be reduced, it will be necessary to develop novel interventions; this topic is currently underresearched, and is an important direction for future work.

One of the most striking conclusions to emerge from the current analyses is that the relative magnitude of genetic and environmental influences appears to be set by the age of 10–12, and that it remains highly stable thereafter, both in terms of the etiology of language, decoding and reading comprehension individually, and also in terms of their interrelationships. While the complexity of the spoken and written language that children use continues to develop throughout adolescence (Nippold, 1998), the contribution of underlying genetic influences driving individual differences in these skills seems to stabilize at a relatively early point.

Two points concerning the measures merit noting. First, with respect to language, the current analyses use a diverse range of measures, which include vocabulary but also extend to receptive grammar, figurative language, and inference making. Despite this diversity, the pattern of association with reading remains constant across these measures, and is also consistent with previous work focusing specifically on measures of listening comprehension (Harlaar et al., 2010; Keenan et al., 2006) and vocabulary (Harlaar et al., 2010; Olson et al., 2011). This suggests that variations in general language ability, rather than a specific aspect of language, are relevant to individual variation in reading skill.

A second, similar point can be made about reading fluency. We used two quite different measures to assess this construct, which tested both word-level and sentence-level reading. The Woodcock-Johnson Reading Fluency test that we used at ages 12 and 16 requires children to comprehend each sentence in order to decide whether or not it describes a true statement, and conceivably this might have inflated the relationship between our reading fluency and reading comprehension constructs. That is, we may have underestimated the dissociation between reading fluency and comprehension. However, we think this is unlikely, because the phenotypic, genetic and environmental relationships are virtually identical whether or not we include the Woodcock-Johnson measure at 12, the age at which we have both the TOWRE and the WJ (details available from the corresponding author). In addition, the TOWRE, which we used at 7 and 12, arguably relies more heavily on decoding skills in younger children than it does at older ages. Given this, it was all the more striking that individual differences in this construct were as stable as they were, both in terms of the high and unchanging heritability across ages, and in its multivariate relationships to oral language and reading comprehension.

While the current study is unique in that it offers a longitudinal perspective on reading development over an unusually long time-frame, from early reading through to mid-adolescence, it also has some significant limitations which must be borne in mind when interpreting the results. One of these is the lack of reading comprehension data in our sample at age 7. There is strong evidence from the behavioral literature that initially, reading comprehension is heavily reliant on decoding skills, and that as decoding skills improve, reading comprehension draws more heavily on oral language competence (Catts, Hogan, & Adlof, 2005; Gough, Hoover, & Peterson, 1996). This picture is supported by behavioral genetic evidence from the ILTS, showing that genetic influences on reading comprehension at age 7 overlap entirely with those for decoding, but that these can be dissociated by the age of 10 (Byrne et al., 2006; Olson et al., 2011). We cannot use our data to replicate this finding, and specifically to pinpoint when the picture we see so clearly at ages 12 and 16 first emerges, of reading comprehension patterning with oral language rather than decoding.

A second limitation concerns the single measures for oral language (expressive vocabulary) at age 7, and for reading fluency and comprehension at age 16. It would be preferable in terms of psychometric reliability to have multiple measures for each construct at each time point; multiple measures would have also allowed for the use of latent factors in the longitudinal analyses, which mitigate against measurement error. However, all three of these measures have acceptable-to-good internal consistency and test–retest reliability, and also relatively high MZ cross-twin correlations (see Table 3), which is an additional indicator of reliability. Previous work incorporating the 7-year vocabulary measure yielded similar results to a global teacher rating of speaking and listening skills (Hayiou-Thomas, Dale, & Plomin, 2012), and the high phenotypic and genetic correlations between 12- and 16-year reading measures are also reassuring in terms of measure validity. Thus, we think it is unlikely that the pattern of results would have looked very different had resources allowed for multiple measures for each construct, at all assessments waves.

In the current article, we have attempted to capture the longitudinal relationships among three constructs—oral language, reading fluency, and reading comprehension—in two ways: by presenting the longitudinal trajectories of each construct separately, and the multivariate relationships among them across ages. It was not computationally feasible to combine these into a single model. A valuable future direction would be to focus on the dynamics of these developmental relationships, potentially through the use of cross-lagged models, in order to shed light on how early variation in one construct (e.g., reading fluency) may drive later variation in a second construct (e.g., vocabulary). A clear picture of how these relationships change over the course of development within the normal range of ability would provide an important context for examining potential “bottlenecks” in development, as they relate to language-learning difficulties.

## Supplementary Material

10.1037/dev0000297.supp

## Figures and Tables

**Table 1 tbl1:** Means, Standard Deviations, and ANOVA Results by Sex and Zygosity for Reading and Language Measures at 7, 12, and 16 Years

Constructs	All		MZ		DZ		Female		Male		Sex		Zygosity		Zyg.*Sex		Tot.
	*M (N)*	*SD*	*M (N)*	*SD*	*M (N)*	*SD*	*M (N)*	*SD*	*M (N)*	*SD*	*p*	η^*2*^	*p*	η^*2*^	*p*	η^*2*^	*R*^*2*^
Reading fluency 7	.00 (*N* = 4,856)	1.00	−.05 (*N* = 1,731)	1.00	.03 (*N* = 3,125)	1.00	.05 (*N* = 2,517)	.97	−.05 (*N* = 2,339)	1.04	.00	.00	.00	.00	.06	.00	.004
Language 7	.02 (*N* = 3,867)	.95	−.04 (*N* = 1,384)	.92	.05 (*N* = 2,483)	.97	.01 (*N* = 1,997)	.96	.03 (*N* = 1,870)	.95	.38	.00	.01	.00	.23	.00	.002
Reading fluency 12	.01 (*N* = 5,154)	.97	−.05 (*N* = 1,873)	.96	.05 (*N* = 3,281)	.97	.05 (*N* = 2,800)	.97	−.03 (*N* = 2,354)	.97	.00	.00	.00	.00	.68	.00	.004
Reading compr. 12	.03 (*N* = 5,136)	.97	−.03 (*N* = 1,858)	.98	.06 (*N* = 3,278)	.96	.02 (*N* = 2,785)	.94	.04 (*N* = 2,351)	1.00	.27	.00	.00	.00	.21	.00	.002
Language 12	.01 (*N* = 4,347)	.99	−.03 (*N* = 1,600)	.98	.04 (*N* = 2,747)	.99	.02 (*N* = 2,420)	.99	.01 (*N* = 1,927)	.98	.96	.00	.04	.00	.42	.00	.001
Reading fluency 16	.00 (*N* = 2,355)	.99	−.06 (*N* = 874)	.96	.03 (*N* = 1,481)	1.00	.04 (*N* = 1,382)	.98	−.06 (*N* = 973)	1.01	.03	.00	.05	.00	.29	.00	.004
Reading compr. 16	.02 (*N* = 1,932)	.98	−.01 (*N* = 730)	1.00	.04 (*N* = 1,202)	.96	−.01 (*N* = 1,166)	.98	.07 (*N* = 766)	.97	.12	.00	.34	.01	.99	.00	.000
Language 16	.01 (*N* = 2,491)	.96	−.03 (*N* = 921)	.95	.04 (*N* = 1,570)	.97	.03 (*N* = 1,449)	.96	−.01 (*N* = 1,042)	.98	.24	.00	.04	.00	.80	.00	.001
*Note.* *M* = mean; *SD* = Standard deviation; *N* = sample size; MZ = Monozygotic twins; DZ = Dizygotic twins; Sex = *p*-value for sex effect; Zyg. = *p*-value for zygosity effect; Sex*Zyg. = *p*-value for sex by zygosity interaction; R^2^ = proportion of total variance accounted for by sex and zygosity. ANOVAs performed using one randomly selected twin in each pair. The scores have been corrected for age and cleared of outliers ±3 standard deviations.																	

**Table 2 tbl2:** Phenotypic Correlations (and N) Between Oral language, Reading Fluency, and Reading Comprehension Measures at Ages 7, 12, and 16

Constructs	1	2	3	4	5	6	7	8
1. Language at 7	1							
	(3867)							
2. Language at 12	.45**	1						
	(2100)	(4347)						
3. Language at 16	.42**	.58**	1					
	(1729)	(1732)	(2491)					
4. Reading fluency at 7	.39**	.40**	.41**	1				
	(3806)	(2504)	(2055)	(4856)				
5. Reading fluency at 12	.35**	.41**	.44**	.70**	1			
	(2505)	(4241)	(2009)	(3030)	(5154)			
6. Reading fluency at 16	.30**	.40**	.43**	.55**	.64**	1		
	(1637)	(1639)	(2338)	(1944)	(1903)	(2355)		
7. Reading comp. at 12	.37**	.62**	.56**	.44**	.45**	.43**	1	
	(2462)	(4120)	(1949)	(2975)	(4988)	(1848)	(5136)	
8. Reading comp. at 16	.34**	.49**	.57**	.34**	.37**	.37**	.49**	1
	(1386)	(1400)	(1928)	(1629)	(1619)	(1859)	(1568)	(1932)
*Note.* The variables are corrected for age and outliers outside ±3 standard deviations removed. Sample size is in brackets below each correlation coefficient.								
** *p* < .01.								

**Table 3 tbl3:** Intraclass Correlations and Heritability Parameter Estimates From Univariate Models

Constructs	Intraclass correlations		Parameter estimated from univariate model fitting		
	MZ [95% CI]	DZ [95% CI]	h^2^ [95% CI]	c^2^ [95% CI]	e^2^ [95% CI]
Oral language					
Age 7	.62 [.59, .66] *N* = 1,320	.50 [.47, .53] *N* = 2,372	.27 [.19, .35]	.37 [.30, .43]	.36 [.34, .39]
Age 12	.68 [.65, .71] *N* = 1,537	.44 [.41, .48] *N* = 2,577	.47 [.40, .54]	.22 [.15, .28]	.31 [.29, .34]
Age 16	.62 [.58, .66] *N* = 854	.36 [.32, .41] *N* = 1,397	.55 [.44, .66]	.09 [.00, .18]	.36 [.22, .39]
Reading fluency					
Age 7	.85 [.84, .87] *N* = 1,704	.48 [.46, .51] *N* = 3,078	.73 [.68, .78]	.12 [.07, .17]	.15 [.14, .16]
Age 12	.77 [.75, .78] *N* = 1,831	.41 [.38, .44] *N* = 3,133	.71 [.51, .76]	.06 [.00, .16]	.23 [.02, .25]
Age 16	.67 [.63, .71] *N* = 782	.36 [.31, .41] *N* = 1,277	.64 [.53, .71]	.04 [.00, .12]	.32 [.29, .35]
Reading comprehension					
Age 12	.62 [.59, .64] *N* = 1,791	.40 [.37, .42] *N* = 3,108	.44 [.36, .51]	.18 [.12, .24]	.38 [.36, .41]
Age 16	.50 [.43, .55] *N* = 647	.23 [.17, .29] *N* = 984	.51 [.45, .55]	.00 [.00, .12]	.49 [.45, .55]
*Note*. *N* = number of twin pairs contributing to the correlation. The heritability estimates report the parameters from the full ACE model (Table 2, online supplementary material).					

**Table 4 tbl4:** Genetic and Environmental Correlations (with 95% Confidence Intervals) from Age-to-Age, for (a) Oral Language, (b) Reading Fluency, and (c) Reading Comprehension

Constructs	Genetic correlations		Shared environmental correlations		Unique environmental correlations	
	Language: Age 7	Language: Age 12	Language: Age 7	Language: Age 12	Language: Age 7	Language: Age 12
Language: Age 12	.63 [.44, .83]		.69 [.51, .84]		.09 [.01, .15]	
Language: Age 16	.63 [.44, .85]	.89 [.75, .93]	.80 [.50, 1.00]	.99 [.70, 1.00]	.07 [−.06, .14]	.14 [.06, .21]
	Reading fluency: Age 7	Reading fluency: Age 12	Reading fluency: Age 7	Reading fluency: Age 12	Reading fluency: Age 7	Reading fluency: Age 12
Reading fluency: Age 12	.84 [.80, .88]		.58 [.15, .85]		.30 [.25, .35]	
Reading fluency: Age 16	.72 [.65, .80]	.78 [.73, .86]	.64 [.00, 1.00]	.94 [.23, 1.00]	.13 [.06, .20]	.26 [.18, .32]
	Reading comprehension: Age 16					
Reading comprehension: Age 12	.83 [.71, 1.00]		1.00 [−1.00, 1.00]		.12 [.04, .20]	

**Table 5 tbl5:** Genetic and Environmental Correlations (with 95% Confidence Intervals) Between (a) Oral Language and Reading Fluency at Age 7, (b) Oral Language, Reading Fluency, and Reading Comprehension at Age 12, and (c) Age 16

	Language					
Age 7	Genetic correlation		Shared environmental correlation		Unique environmental correlation	
Reading fluency	.47 [.37, .58]		.79 [.60, 1.00]		.09 [.04, .15]	
Age 12	Language	Reading fluency	Language	Reading fluency	Language	Reading fluency
Reading fluency	.46 [.40, .53]		.93 [.66, 1.00]		.14 [.09, .18]	
Reading comprehension	.81 [.75, .89]	.58 [.51, .65]	.95 [.81, 1.00]	.77 [.45, 1.00]	.21 [.17, .26]	.16 [.12, .21]
Age 16	Language	Reading fluency	Language	Reading fluency	Language	Reading fluency
Reading fluency	.58 [.50, .69]		1.00 [−1.00, 1.00]		.07 [.01, .14]	
Reading comprehension	.87 [.80, 1.00]	.58 [.50, .73]	1.00 [−1.00, 1.00]	1.00 [−1.00, 1.00]	.21 [.00, .62]	.07 [.01, .14]

**Table 6 tbl6:** Summary of Model Fitting Results for Nested Common Pathway Models

Model-Base comparison	Model	−2LL	*df*	(Δ−2LL)	AIC	BIC	*p*-value	Parameters
1. Cholesky ACE	—	140275.80	60112	—	20051.82	−405479.71	—	116
2. Cholesky ACE	Comm.P_1 Factor	143052.40	60188	2776.63	22680.45	−403393.08	<.0001	43
3. **Cholesky ACE**	**Comm.P_2 Factor**	**140714.30**	**60185**	**438.45**	**20344.27**	**−405704.02**	**<.0001**	**49**
4. Cholesky ACE	Comm.P_3 Factor	140691.10	60173	415.29	20345.11	−405672.71	<.0001	58
5. Comm.P_1 Factor	Comm.P_2 Factor	140714.30	60185	2338.18	20344.27	−405704.02	<.0001	49
6. Comm.P_3 Factor	Comm.P_2 Factor	140714.30	60181	23.17	20344.27	−405704.02	.003	49
*Note*. Comm.P_1 Factor = Common pathway 1-factor model, the indices P_2 and P_3 indicate models with 2 and 3 latent factors respectively; Δ−2LL = Difference in likelihood between the Model Basel comparison and the model in the second column; *p*-values = significance in likelihood difference between the Model Base and the Model; AIC = Akaike Information Criterion; BIC = Bayesian Information Criterion; −2LL reports = minus 2 likelihood. The parameters estimated are reported for the model in the second column. Model comparison for Lines 2, 3 and 4 is between the Cholesky ACE and the nested Common pathway 1-factor, 2-factor, and 3-factor models. In Line 5 the comparison is between the 1-factor and 2-factor models, in Line 6, the comparison is between the 3-factor and 2-factor models. All comparisons show that the models are significantly different from the baseline Cholesky ACE models and each other. The 2-factor solution, which is bolded, yields the smallest AIC and BIC values of all nested models, suggesting that its fit is significantly better than the 1- and 3-factor solutions.								

## Footnotes

1 Note that a three-factor model, with separate factors for reading fluency, reading comprehension, and language, also fit the data reasonably well (though not as well as the more parsimonious two-factor model we present). However, the genetic correlation between language and reading comprehension in this three-factor model was r_g_ = 1.0 (95% CI [.98, 1.0]), indicating essentially complete genetic overlap between these two factors. That is, in terms of interpretation, there is no difference between the two- and three-factor models.

2 We also considered an alternative two-factor model which had a reading factor (fluency and comprehension) and a separate language factor. This model had a significantly worse fit to the data.

## References

[c1] Betjemann R. S., Keenan J. M., Olson R. K., & Defries J. C. (2011). Choice of reading comprehension test influences the outcomes of genetic analyses. Scientific Studies of Reading, 15, 363–382. 10.1080/10888438.2010.493965 21804757 PMC3143485

[c2] Betjemann R. S., Willcutt E. G., Olson R. K., Keenan J. M., DeFries J. C., & Wadsworth S. J. (2007). Word reading and reading comprehension: Stability, overlap and independence. Reading and Writing, 21, 539–558. 10.1007/s11145-007-9076-8

[c3] Blakemore S.-J. (2012). Imaging brain development: The adolescent brain. NeuroImage, 61, 397–406. 10.1016/j.neuroimage.2011.11.080 22178817

[c4] Boker S. M., Neale M. C., Maes H. H., Wilde M. J., Spiegel M., Brick T. R., . . . Brandmaier A. (2012). OpenMx 1.2 user guide. Charlottesville, VA: The OpenMx Project.

[c5] Boker S., Neale M., Maes H., Wilde M., Spiegel M., Brick T., . . . Fox J. (2011). OpenMx: An open source extended structural equation modeling framework. Psychometrika, 76, 306–317. 10.1007/s11336-010-9200-6 23258944 PMC3525063

[c101] Braze D., Mencl W. E., Tabor W., Pugh K. R., Constable R. T., Fulbright R. K., . . . Shankweiler D. P. (2011). Unification of sentence processing via ear and eye: an fMRI study. Cortex: A Journal Devoted to the Study of the Nervous System and Behavior, 47, 416–431. 20117764 10.1016/j.cortex.2009.11.005 PMC2889140

[c6] Byrne B., Coventry W. L., Olson R. K., Samuelsson S., Corley R., Willcutt E. G., . . . Defries J. C. (2009). Genetic and environmental influences on aspects of literacy and language in early childhood: Continuity and change from preschool to Grade 2. Journal of Neurolinguistics, 22, 219–236. 10.1016/j.jneuroling.2008.09.003 20161176 PMC2724015

[c102] Byrne B., Khlentzos D., Olson R. K., & Samuelsson S. (2010). Evolutionary and Genetic Perspectives on Educational Attainment In Littleton K., Wood C., & Kleine Staarman J. (Eds.), International Handbook of Psychology in Education (pp. 3–33). Bingley, UK: Emerald Group Publishing Ltd.

[c7] Byrne B., Samuelsson S., Wadsworth S., Hulslander J., Corley R., DeFries J. C., . . . Olson R. K. (2006). Longitudinal twin study of early literacy development: Preschool through Grade 1. Reading and Writing, 20, 77–102. 10.1007/s11145-006-9019-9

[c8] Catts H. W., Hogan T. P., & Adlof S. M. (2005). Developmental changes in reading and reading difficulties In Catts H. W. & Kamhi A. G. (Eds.), The connections between language and reading difficulties (pp. 25–40). Mahwah, NJ: Erlbaum.

[c9] Chall J. (1983). Stages of reading development. New York, NY: McGraw-Hill.

[c10] Chow B. W-Y., Ho C. S-H., Wong S. W-L., Waye M. M. Y., & Bishop D. V. M. (2011). Genetic and environmental influences on Chinese language and reading abilities. PLoS ONE, 6, e16640 10.1371/journal.pone.0016640 21347359 PMC3037369

[c11] Christopher M. E., Hulslander J., Byrne B., Samuelsson S., Keenan J. M., Pennington B., . . . Olson R. K. (2013a). Modeling the etiology of individual differences in early reading development: Evidence for strong genetic influences. Scientific Studies of Reading, 17, 350–368. 10.1080/10888438.2012.729119 24489459 PMC3905458

[c12] Christopher M. E., Hulslander J., Byrne B., Samuelsson S., Keenan J. M., Pennington B., . . . Olson R. K. (2013b). The genetic and environmental etiologies of individual differences in early reading growth in Australia, the United States, and Scandinavia. Journal of Experimental Child Psychology, 115, 453–467. 10.1016/j.jecp.2013.03.008 23665180 PMC3661747

[c13] Clarke P. J., Snowling M. J., Truelove E., & Hulme C. (2010). Ameliorating children’s reading-comprehension difficulties: A randomized controlled trial. Psychological Science, 21, 1106–1116. 10.1177/0956797610375449 20585051

[c14] Dale P. S., Harlaar N., Hayiou-Thomas M. E., & Plomin R. (2010). The etiology of diverse receptive language skills at 12 years. Journal of Speech, Language, and Hearing Research, 53, 982–992. 10.1044/1092-4388(2009/09-0108) PMC4040409 20605943

[c15] Dale P. S., Harlaar N., & Plomin R. (2005). Correspondence between telephone and teacher assessments of reading: I. Substantial correspondence for a sample of 5,808 children and for extremes. Reading and Writing, 18, 385–400. 10.1007/s11145-004-8130-z

[c16] Farrant B. M., & Zubrick S. R. (2013). Parent–child book reading across early childhood and child vocabulary in the early school years: Findings from the Longitudinal Study of Australian Children. First Language, 33, 280–293. 10.1177/0142723713487617

[c17] Gough P. B., Hoover W. A., & Peterson C. L. (1996). Some observations on a simple view of reading In Cornoldi C. & Oakhill J. (Eds.), Reading comprehension difficulties: Processes and intervention (pp. 1–14). Mahwah, NJ: Erlbaum.

[c18] Hammill D. D., Brown V. L., Larsen S. C., & Wiederholt J. L. (1994). Test of Adolescent and Adult Language (TOAL-3). Austin, TX: Pro-Ed.

[c19] Harlaar N., Cutting L., Deater-Deckard K., Dethorne L. S., Justice L. M., Schatschneider C., . . . Petrill S. A. (2010). Predicting individual differences in reading comprehension: A twin study. Annals of Dyslexia, 60, 265–288. 10.1007/s11881-010-0044-7 20814768 PMC2981603

[c20] Harlaar N., Dale P. S., & Plomin R. (2007). From learning to read to reading to learn: Substantial and stable genetic influence. Child Development, 78, 116–131. 10.1111/j.1467-8624.2007.00988.x 17328696

[c21] Harlaar N., Hayiou-Thomas M. E., Dale P. S., & Plomin R. (2008). Why do preschool language abilities correlate with later reading? A twin study. Journal of Speech, Language, and Hearing Research, 51, 688–705. 10.1044/1092-4388(2008/049) 18506044

[c22] Harlaar N., Spinath F. M., Dale P. S., & Plomin R. (2005). Genetic influences on early word recognition abilities and disabilities: A study of 7-year-old twins. Journal of Child Psychology and Psychiatry, 46, 373–384. 10.1111/j.1469-7610.2004.00358.x 15819646

[c23] Haworth C. M. A., Davis O. S. P., & Plomin R. (2013). Twins Early Development Study (TEDS): A genetically sensitive investigation of cognitive and behavioral development from childhood to young adulthood. Twin Research and Human Genetics, 16, 117–125. 10.1017/thg.2012.91 23110994 PMC3817931

[c24] Haworth C. M. A., Wright M. J., Luciano M., Martin N. G., de Geus E. J. C., van Beijsterveldt C. E. M., . . . Plomin R. (2010). The heritability of general cognitive ability increases linearly from childhood to young adulthood. Molecular Psychiatry, 15, 1112–1120. 10.1038/mp.2009.55 19488046 PMC2889158

[c25] Hayiou-Thomas M. E., Dale P. S., & Plomin R. (2012). The etiology of variation in language skills changes with development: A longitudinal twin study of language from 2 to 12 years. Developmental Science, 15, 233–249. 10.1111/j.1467-7687.2011.01119.x 22356179

[c26] Hayiou-Thomas M. E., Harlaar N., Dale P. S., & Plomin R. (2010). Preschool speech, language skills, and reading at 7, 9, and 10 years: Etiology of the relationship. Journal of Speech, Language, and Hearing Research, 53, 311–332. 10.1044/1092-4388(2009/07-0145) 20360459

[c27] Hayiou-Thomas M. E., Kovas Y., Harlaar N., Plomin R., Bishop D. V. M., & Dale P. S. (2006). Common aetiology for diverse language skills in 4 1/2-year-old twins. Journal of Child Language, 33, 339–368. 10.1017/S0305000906007331 16826830

[c104] Hoff E. (2006). How social contexts support and shape language development. Developmental Review, 26, 55–88.

[c28] Hoover W. A., & Gough P. B. (1990). The simple view of reading. Reading and Writing, 2, 127–160. 10.1007/BF00401799

[c29] Hulslander J., Olson R. K., Willcutt E. G., & Wadsworth S. J. (2010). Longitudinal stability of reading-related skills and their prediction of reading development. Scientific Studies of Reading, 14, 111–136. 10.1080/10888431003604058 20563241 PMC2885806

[c200] Kaplan E., Fein D. C., Kramer J. H., Delis D. & Morris R. (1999). Wechsler Intelligence Scale as a Process Instrumentt: - WISC-III PI. San Antonio, TX: The Psychological Corporation.

[c31] Keenan J. M., Betjemann R. S., Wadsworth S. J., DeFries J. C., & Olson R. K. (2006). Genetic and environmental influences on reading and listening comprehension. Journal of Research in Reading, 29, 75–91. 10.1111/j.1467-9817.2006.00293.x

[c32] Kovas Y., & Plomin R. (2006). Generalist genes: Implications for the cognitive sciences. Trends in Cognitive Sciences, 10, 198–203. 10.1016/j.tics.2006.03.001 16580870

[c33] Logan J. A. R., Hart S. A., Cutting L., Deater-Deckard K., Schatschneider C., & Petrill S. (2013). Reading development in young children: Genetic and environmental influences. Child Development, 84, 2131–2144. 10.1111/cdev.12104 23574275 PMC3773299

[c34] Markwardt F. C. Jr. (1997). Peabody individual achievement test-revised (normative update) manual. Circle Pines, MN: American Guidance Service.

[c103] Martin N. G., & Eaves L. J. (1977). The genetical analysis of covariance structure. Heredity, 38, 79–95. 268313 10.1038/hdy.1977.9

[c35] McGue M., & Bouchard T. J. Jr. (1984). Adjustment of twin data for the effects of age and sex. Behavior Genetics, 14, 325–343. 10.1007/BF01080045 6542356

[c36] Nippold M. A. (1998). Later language development: The school-age and adolescent years (3rd ed.). Austin, TX: Pro-Ed.

[c39] Olson R. K., Keenan J. M., Byrne B., Samuelsson S., Coventry W. L., Corley R., . . . Hulslander J. (2011). Genetic and environmental influences on vocabulary and reading development. Scientific Studies of Reading, 15, 26–46. 10.1080/10888438.2011.536128 21132077 PMC3019615

[c40] Petrill S. A., Deater-Deckard K., Thompson L. A., Schatschneider C., Dethorne L. S., & Vandenbergh D. J. (2007). Longitudinal genetic analysis of early reading: The Western Reserve Reading Project. Reading and Writing, 20, 127–146. 10.1007/s11145-006-9021-2 19829751 PMC2760987

[c41] Plomin R., DeFries J. C., Knopik V. S., & Neiderhiser J. M. (2013). Behavioral genetics (6th ed.). New York, NY: Worth.

[c42] Price T. S., Freeman B., Craig I., Petrill S. A., Ebersole L., & Plomin R. (2000). Infant zygosity can be assigned by parental report questionnaire data. Twin Research, 3, 129–133. 10.1375/136905200320565391 11035484

[c43] Raven J. C., Court J. H., & Raven J. (1998). Mill Hill Vocabulary Scale. Oxford, UK: Oxford Psychologists Press.

[c44] Samuelsson S., Byrne B., Olson R. K., Hulslander J., Wadsworth S., Corley R., . . . Defries J. C. (2008). Response to early literacy instruction in the United States, Australia, and Scandinavia: A behavioral-genetic analysis. Learning and Individual Differences, 18, 289–295. 10.1016/j.lindif.2008.03.004 19122888 PMC2570222

[c45] Sénéchal M., Pagan S., Lever R., & Ouellette G. P. (2008). Relations among the frequency of shared reading and 4-year-old children’s vocabulary, morphological and syntax comprehension, and narrative skills. Early Education and Development, 19, 27–44. 10.1080/10409280701838710

[c46] Snowling M. J., Stothard S. E., Clarke P., Bowyer-Crane C., Harrington A., Truelove E., . . . Hulme C. (2009). York assessment of reading for comprehension—Passage reading. London, UK: GL Assessment.

[c47] Spinath F. M., Price T. S., Dale P. S., & Plomin R. (2004). The genetic and environmental origins of language disability and ability. Child Development, 75, 445–454. 10.1111/j.1467-8624.2004.00685.x 15056198

[c48] Taylor J., & Schatschneider C. (2010). Genetic influence on literacy constructs in kindergarten and first grade: Evidence from a diverse twin sample. Behavior Genetics, 40, 591–602. 10.1007/s10519-010-9368-7 20563747 PMC3529359

[c49] Torgesen J. K., Wagner R. K., & Rashotte C. A. (1999). Test of Word Reading Efficiency (TOWRE). Austin, TX: Pro-ed.

[c50] van den Bos K. P., Zijlstra B. J., & Lutje Spelberg H. C. (2002). Life-span data on continuous-naming speeds of numbers, letters, colors, and pictured objects, and word-reading speed. Scientific Studies of Reading, 6, 25–49. 10.1207/S1532799XSSR0601_02

[c51] Wechsler D. (1992). Wechsler Intelligence Scale for Children manual (3rd ed., UK; WISC-III-UK). London, UK: Psychological Corporation.

[c52] Wiig E. H., Secord W., & Sabers D. (1989). Test of language competence (expanded ed.). San Antonio, TX: Psychological Corporation.

[c53] Woodcock R. W., McGrew K. S., & Mather N. (2001). Woodcock-Johnson III. Itasca, IL: Riverside Publishing.

