# Supplementary material for: The Genetic Architecture of Oral Language, Reading Fluency, and Reading Comprehension: A Twin Study From 7 to 16 Years
Source: Dev Psychol. 2017 Jun;53(6):1115–29. doi: 10.1037/dev0000297 (PMC5444555; doi:10.1037/dev0000297)
Supplement: Supplementary file 1 [file z2p999173848so1.docx]

**Supplemental Materials**

**The Genetic Architecture of Oral Language, Reading Fluency, and Reading Comprehension: A Twin Study From 7 to 16 Years**

**by M. G. Tosto et al., 2017, *Developmental Psychology***

**http://dx.doi.org/10.1037/dev0000297**

| Table 1: Model fitting parameters for correlated factors models at each age. Age 7: Language & Reading Fluency; Ages 12 and 16: Language, Reading Fluency and Reading Comprehension.   \| Age \| Model \| **-2LL** \| **df** \| **(Δ-2LL)** \| **AIC** \| **BIC** \| p-value Model-base and Model \| **ep** \| \| --- \| --- \| --- \| --- \| --- \| --- \| --- \| --- \| --- \| \| 7 \| Saturated \| 43244.71 \| 17387 \|  \| 8470.71 \| -111468.80 \|  \| 28 \| \|  \| Multivariate ACE \| 43270.74 \| 17404 \| 26.03 \| 8462.74 \| -111594.04 \| .07 \| 11 \| \| 12 \| Saturated \| 70665.44 \| 29163 \|  \| 12339.44 \| -186936.33 \|  \| 54 \| \|  \| Multivariate ACE \| 70708.83 \| 29196 \| 43.39 \| 12316.83 \| -187184.43 \| .11 \| 21 \| \| 16 \| Saturated \| 34282.50 \| 13542 \|  \| 7198.50 \| -84998.41 \|  \| 54 \| \|  \| Multivariate ACE \| 34318.21 \| 13575 \| 35.71 \| 7168.21 \| -85253.37 \| .34 \| 21 \| \|  \|  \|  \|  \|  \|  \|  \|  \|  \| |
| --- | --- | --- | --- | --- | --- | --- | --- | --- | --- | --- | --- | --- | --- | --- | --- | --- | --- | --- | --- | --- | --- | --- | --- | --- | --- | --- | --- | --- | --- | --- | --- | --- | --- | --- | --- | --- | --- | --- | --- | --- | --- | --- | --- | --- | --- | --- | --- | --- | --- | --- | --- | --- | --- | --- | --- | --- | --- | --- | --- | --- | --- | --- | --- | --- | --- | --- | --- | --- | --- | --- | --- | --- |

| Table 2: Univariate model fitting and parameters estimated from the univariate models, at age 7 (Language and Reading Fluency), age 12 and age 16 (Language, Reading Fluency, Reading Comprehension) | | | | | | | | | | | | |
| --- | --- | --- | --- | --- | --- | --- | --- | --- | --- | --- | --- | --- |
| Measures used in the model | **Model** | **-2LL** | **df** | **(Δ-2LL)** | **BIC** | **p** | **ep** | | **Obs.Stat** | **h^2^ (95%CI)** | **c^2^ (95%CI)** | **e^2^ (95%CI)** |
| *AGE 7* |  |  |  |  |  |  | |  |  |  |  |  |
|  |  |  |  |  |  |  | |  |  |  |  |  |
| *Reading Fluency* | *Saturated* | 24479.34 | 9694 | - | -61780.09 | -- | | 10 | 9704 | - | - | - |
|  | ***ACE*** | **24490.01** | **9700** | **10.67** | **-61822.81** | **.10** | | **4** | **9704** | **.73 (.68; .78)** | **.12 (.07; .17)** | **.15 (.14; .16)** |
|  | *AE* | 24509.35 | 9701 | 19.34 | -61812.37 | .00 | | 3 | 9704 | .85 (.84; .86) | - | .15 (.14; .16) |
|  | *CE* | 25232.59 | 9701 | 742.58 | -61089.13 | .00 | | 3 | 9704 | - | .62 (.60; .64) | .38 (.36; .40) |
|  |  |  |  |  |  |  | |  |  |  |  |  |
| *Language* | *Saturated* | 19791.30 | 7701 | - | -48733.96 | - | | 10 | 7711 |  |  |  |
|  | ***ACE*** | **19805.42** | **7707** | **14.12** | **-48773.23** | **.03** | | **4** | **7711** | **.27 (.19; .35)** | **.37 (.30; .43)** | **.36 (.34; .39)** |
|  | *AE* | 19915.02 | 7708 | 109.59 | -48672.53 | .00 | | 3 | 7711 | .67 (.65; .69) | - | .33(.31; .35) |
|  | *CE* | 19848.90 | 7708 | 43.47 | -48738.65 | .00 | | 3 | 7711 | - | .55 (.53 .57) | .45 (.43; .47) |
|  |  |  |  |  |  |  | |  |  |  |  |  |
| *AGE 12* |  |  |  |  |  |  | |  |  |  |  |  |
|  |  |  |  |  |  |  | |  |  |  |  |  |
| *Language* | *Saturated* | 23030.72 | 8696 | - | -53782.54 | - | | 10 | 8706 |  |  |  |
|  | ***ACE*** | **23038.39** | **8702** | **7.67** | **-53827.86** | **.26** | | **4** | **8706** | **.47 (.40; .54)** | **.22 (.15; .28)** | **.31 (.29; .34)** |
|  | *AE* | 23079.51 | 8703 | 41.12 | -53795.58 | .00 | | 3 | 8706 | .71 (.81; .82) | - | .29 (.28; .32) |
|  | *CE* | 23188.86 | 8703 | 150.47 | -53686.22 | .00 | | 3 | 8706 | - | .54 (.52 .56) | .46 (.44; .48) |
|  |  |  |  |  |  |  | |  |  |  |  |  |
| *Reading Fluency* | *Saturated* | 26407.59 | 10269 | - | -64300.24 | - | | 10 | 10279 | - | - | - |
|  | *ACE* | 26422.67 | 10275 | 15.08 | -64338.16 | .02 | | 4 | 10279 | .71(.51; .76) | .06 (.00; .16) | .23(.02; .25) |
|  | ***AE*** | **26426.23** | **10276** | **3.56** | **-64343.44** | **.06** | | **3** | **10279** | **.77 (.76; .79)** | **-** | **.23 (.21; .24** |
|  | *CE* | 26899.01 | 10276 | 476.34 | -63870.66 | .00 | | 3 | 10279 | - | .54 (.53; .57) | .46 (.43; .47) |
|  |  |  |  |  |  |  | |  |  |  |  |  |
| *Reading Compreh.* | *Saturated* | 26993.09 | 10222 | - | -63299.59 | - | | 10 | 10232 | - | - | - |
|  | ***ACE*** | **27008.77** | **10228** | **15.68** | **-63336.91** | **.02** | | **4** | **10232** | **.44 (.36; .51)** | **.18 (.12; .24** | **.38 (.36; .41)** |
|  | *AE* | 27039.77 | 10229 | 31.00 | -63314.74 | .00 | | 3 | 10232 | .64(.62; .66) | - | .36 (.34; .38 |
|  | *CE* | 27131.47 | 10229 | 122.70 | -63223.04 | .00 | | 3 | 10232 | - | .48(.46; .50) | .42 (.50; .54) |
|  |  |  |  |  |  |  | |  |  |  |  |  |
| *AGE 16* |  |  |  |  |  |  | |  |  |  |  |  |
|  |  |  |  |  |  |  | |  |  |  |  |  |
| *Language* | *Saturated* | 13279.64 | 4991 | - | -30682.18 | - | | 10 | 5001 | - | - | - |
|  | *ACE* | 13287.29 | 4997 | 7.65 | -30727.39 | .27 | | 4 | 8558 | .55 (.44; 66) | .09 (.00; .18) | .36(.22; .39) |
|  | ***AE*** | **13290.95** | **4998** | **3.66** | **-30732.54** | **.06** | | **3** | **8558** | **.65 (.62; 68)** | **-** | **.35(.31; .38)** |
|  | *CE* | 13375.03 | 4998 | 87.74 | -30648.45 | .00 | | 3 | 8558 | - | .47(.43; .50) | .53 (.50; .56) |
|  |  |  |  |  |  |  | |  |  |  |  |  |
| *Reading Fluency* | *Saturated* | 12751.30 | 4722 | - | -28841.11 | - | | 10 | 4732 | - | - | - |
|  | *ACE* | 12761.17 | 4728 | 9.87 | -28884.09 | .13 | | 4 | 4732 | .64(.53; .71) | .04 (.00; .12) | .32(.29; .36) |
|  | ***AE*** | **12761.65** | **4729** | **0.48** | **-28892.42** | **.17** | | **3** | 4732 | **.68 (.65; .71)** | **-** | **.32(.29; .35)** |
|  | *CE* | 12880.41 | 4729 | 119.24 | -28773.66 | .00 | | 3 | 4732 | - | .48 (.44; .51) | .42 (.49; .56) |
|  |  |  |  |  |  |  | |  |  |  |  |  |
| *Reading Compreh.* | *Saturated* | 10556.75 | 3853 | - | -23381.32 | - | | 10 | 3863 | - | - | - |
|  | *ACE* | 10564.42 | 3859 | 7.67 | -23426.50 | .26 | | 4 | 3863 | .51(.45; .55) | .00 (.00; .12) | .49(.45; .55) |
|  | ***AE*** | **10564.42** | **3860** | **0.00** | **-23435.31** | **1.00** | | **3** | **3863** | **.51(.45; .55)** | **-** | **.49(.45; .55)** |
|  | *CE* | 10602.17 | 3860 | 37.75 | -23397.56 | .00 | | 3 | 3863 | - | .35 (.31; .40) | .65 (.60; .69) |
| Note: Δ-2LL = Difference in likelihood between the Saturated and nested ACE, AE and CE models. The AE and CE nested models are obtained by dropping the C and A parameters respectively. p = p-value, denotes significance in likelihood difference between the Saturated with ACE and the ACE model with the nested models. ep = estimated parameter. BIC = Bayesian Information Criterion; smaller values indicate a model with a better fit. -2LL = minus 2 log likelihood. Obs.Stat. = Observed statistics. h^2^, c^2^, e^2^, respectively represent the estimates for: genetic, shared environmental and non-shared environmental contribution to individual differences in the measure of interest. 95% Confidence interval for each estimate are in parenthesis. The best fitting model is noted with bold characters. In the main manuscript, all reported heritability estimates refer to the ACE model, irrespective of the most parsimonious | | | | | | | | | | | | |

| Table 3: Multivariate Model Fitting on longitudinal measures of Language, Reading Fluency and Reading Comprehension | | | | | | | | | |
| --- | --- | --- | --- | --- | --- | --- | --- | --- | --- |
| **Measure used in the model** | **Model** | **-2LL** | **df** | **(Δ-2LL)** | **AIC** | **BIC** | **p-value** | **ep** | **Obs. Stat.** |
| Language measures age 7, 12, 16 | *Saturated* | 53762.33 | 21364 | - | 11034.33 | -140200.96 | -- | 54 | 21418 |
|  | *Multi-ACE* | 53798.87 | 21397 | 36.54 | 11004.87 | -140464.03 | .31 | 21 | 21418 |
|  |  |  |  |  |  |  |  |  |  |
| Reading Fluency measures age 7, 12, 16 | *Saturated* | 58118.80 | 24661 | - | 8796.80 | -165777.88 | -- | 54 | 24715 |
|  | *Multi-ACE* | 58165.25 | 24694 | 46.45 | 8777.25 | -166031.04 | 0.06 | 21 | 24715 |
|  |  |  |  |  |  |  |  |  |  |
| Reading Comprehension measures age 12 and 16 | *Saturated* | 36743.70 | 14067 | - | 8609.70 | -90970.29 | -- | 28 | 14095 |
|  | *Multi-ACE* | 36772.24 | 14084 | 28.55 | 8604.24 | -91096.08 | 0.04 | 11 | 14095 |
| Note: Multi-ACE = Multivariate model obtained using a Cholesky decomposition. Δ-2LL = Difference in likelihood between the Saturated and Multi-ACE models. p-value = denote significance in likelihood difference between the Saturated and Multi-ACE models. ep = estimated parameters AIC = Akaike Information Criterion. BIC = Bayesian Information Criterion. -2LL = minus 2 log likelihood. Obs.Stat. = Observed statistics. The lower AIC and BIC indices in the 3 Multi-ACE models compared to the respective Saturated models suggest a good fit of all multivariate models. For the models that use Language and Reading Fluency measures, the multivariate models are not significantly different from the respective Saturated models (p-value > .05). In the longitudinal analysis of Reading Comprehension, the multivariate model is significantly better than the Saturated (p-value < .04). The genetic, shared-environmental and non shared environmental correlations derived from this model are detailed in the results section of the main manuscript. The value of the path-coefficients indexing shared and specific genetic and environmental influences on the measures over time, are reported in Figures 1a, 1b and 1c. | | | | | | | | | |

| **Table 4: Univariate sex-limitation model fitting Language and Reading Fluency at age 7, 12, 16. For Reading Comprehension age 12 and 16** | | | | | | | | | | | | | |
| --- | --- | --- | --- | --- | --- | --- | --- | --- | --- | --- | --- | --- | --- |
| **Measure** | **Model** | **-2LL** | **df** | **(Δ-2LL)** | **AIC** | **BIC** | **p (value)** | **ep** |  | h^2^ (95%CI) | c^2^ (95%CI) | e^2^ (95%CI) | DZos correlations |
| **Language age 7** |  |  |  |  |  |  |  |  |  |  |  |  |  |
|  | *Full sex-limitation model* | 13093.45 | 5123 |  | 2847.45 | -38099.09 | n/a | 9 | 5132 | males .17 (.03; .33) | males .45 (.32; .57) | males .38 (.36; .44) | r_g_= .50 (.05; .50) |
|  |  |  |  |  |  |  |  |  |  | females .27 (.15; .39) | females .35 (.25; .46) | females .38 (.33; .45) | r_c_=1.0 (.80; 1.0) |
|  | *Common Effects model* | 13093.45 | 5124 | 0.00 | 2845.45 | -38109.08 | 1.00 | 8 | 5132 | males .17 (.03; .33) | males .45 (.32; .57) | males .38 (.36; .44) |  |
|  |  |  |  |  |  |  |  |  |  | females .27 (.15; .39) | females .35 (.25; .46) | females .38 (.33; .45) |  |
|  | *Scalar Effects model* | 13094.67 | 5126 | 1.21 | 2842.67 | -38127.85 | .54 | 6 | 5132 |  |  |  |  |
|  |  |  |  |  |  |  |  |  |  |  |  |  |  |
|  | *Null model* | 13094.67 | 5127 | 0.00 | 2840.67 | -38137.84 | .99 | 5 | 5132 | all .23 (.20; .32) | all .39 (.31; .47) | all .38 (.35; .42) |  |
|  |  |  |  |  |  |  |  |  |  |  |  |  |  |
| **Reading Fluency age 7** |  |  |  |  |  |  |  |  |  |  |  |  |  |
|  | *Full sex-limitation model* | 15377.58 | 6277 |  | 2823.59 | -47346.52 |  | 9 | 6286 | males .57 (.46; .69) | males .27 (.16; .38) | males .16 (.14; .18) | r_g_= .28 (.12; .42) |
|  |  |  |  |  |  |  |  |  |  | females .67 (.56; .78) | females .17 (.06; .27) | females .16 (.14; .18) | r_c_=.38 (.01; .69) |
|  | *Common Effects model* | 15385.36 | 6278 | 7.77 | 2829.36 | -47348.74 | .01 | 8 | 6286 | males .55 (.45; .81) | males .29 (.03; .39) | males .16 (.14; .18) |  |
|  |  |  |  |  |  |  |  |  |  | females .82 (.65; .85) | females .02 (.00; .18) | females .16 (.14; .18) |  |
|  | *Scalar Effects model* | 15390.71 | 6280 | 1.21 | 2830.71 | -47363.37 | .07 | 6 | 6286 |  |  |  |  |
|  |  |  |  |  |  |  |  |  |  |  |  |  |  |
|  | *Null model* | 15407.06 | 6281 | 16.35 | 2845.06 | -47357.02 | .00 | 5 | 6286 | all .71 (.65; .79) | all .13 (.12; .19) | all .16 (.14; .17) |  |
|  |  |  |  |  |  |  |  |  |  |  |  |  |  |
| **Language age 12** |  |  |  |  |  |  |  |  |  |  |  |  |  |
|  | *Full sex-limitation model* | 17477.88 | 6676 |  | 4125.88 | -49233.31 |  | 9 | 6685 | males .44 (.31; .57) | males .24 (.12; .36) | males .32 (.28; .36) | r_g_= .50 (.37; .50) |
|  |  |  |  |  |  |  |  |  |  | females .54 (.43; .64) | females .17 (.08; .26) | females .29 (.27; .33) | r_c_=1.0 (.73; .1.0) |
|  | *Common Effects model* | 17477.88 | 6677 | 0.00 | 4123.88 | -49243.30 | 1.00 | 8 | 6685 | males .44 (.31; .57) | males .24 (.12; .36) | males .32 (.28; .36) |  |
|  |  |  |  |  |  |  |  |  |  | females .54 (.43; .64) | females .17 (.08; .26) | females .29 (.27; .33) |  |
|  | *Scalar Effects model* | 17479.59 | 6679 | 1.72 | 4121.59 | -49261.57 | .42 | 6 | 6685 |  |  |  |  |
|  |  |  |  |  |  |  |  |  |  |  |  |  |  |
|  | *Null model* | 17479.59 | 6680 | 1.18 | 4119.59 | -49271.57 | .99 | 5 | 6685 | all .51 (.42; .58) | all .19 (.12; .26) | all .30 (.28; .33) |  |
|  |  |  |  |  |  |  |  |  |  |  |  |  |  |
| **Reading Fluency age 12** |  |  |  |  |  |  |  |  |  |  |  |  |  |
|  | *Full sex-limitation model* | 19662.86 | 7799 |  | 4064.86 | -58270.11 |  | 9 | 7808 | males .77(.65; .82) | males .01 (.00; .12) | males .22 (.20; .25) | r_g_= .50 (.00; .50) |
|  |  |  |  |  |  |  |  |  |  | females .59 (.48; .70) | females .17 (.07; .27) | females .24 (.22; .26) | r_c_= 1.0 (.00; 1.0) |
|  | *Common Effects model* | 19662.86 | 7800 | 0.00 | 4062.86 | -58280.10 | .00 | 8 | 7808 | males .77 (.70; .80) | males .01 (.00; .07) | males .22 (.20; .25) |  |
|  |  |  |  |  |  |  |  |  |  | females .59 (.48; .70) | females .17 (.07; .27) | females .24 (.22; .26) |  |
|  | *Scalar Effects model* | 19668.94 | 7802 | 6.07 | 4064.94 | -58294.02 | .05 | 6 | 7808 |  |  |  |  |
|  |  |  |  |  |  |  |  |  |  |  |  |  |  |
|  | *Null model* | 19670.73 | 7803 | 16.35 | 4064.74 | -58302.21 | .18 | 5 | 7808 | all .71 (.35; .78) | all .06 (.00; .13) | all .23(.21; .25) |  |
|  |  |  |  |  |  |  |  |  |  |  |  |  |  |
| **Reading Comprehension age 12** |  |  |  |  |  |  |  |  |  |  |  |  |  |
|  | *Full sex-limitation model* | 20006.34 | 7714 |  | 4578.34 | -57077.26 |  | 9 | 7723 | males .48 (.36; .58) | males .15 (.07; .25) | males .37 (.33; .41) | r_g_= .50 (.26; .50) |
|  |  |  |  |  |  |  |  |  |  | females .32 (.20; .44) | females .30 (.19; .40) | females .38 (.35; .42) | r_c_= 1.0 (.68; 1.0) |
|  | *Common Effects model* | 20006.34 | 7715 | 0 | 4576.34 | -57087.25 | 1.0 | 8 | 7723 | males .48 (.36; .58) | males .15 (.07; .25) | males .37 (.33; .41) |  |
|  |  |  |  |  |  |  |  |  |  | females .32 (.20; .44) | females .30 (.19; .40) | females .38 (.35; .42) |  |
|  | *Scalar Effects model* | 20023.11 | 7717 | 16.76 | 4589.11 | -57090.47 | .00 | 6 | 7723 |  |  |  |  |
|  |  |  |  |  |  |  |  |  |  |  |  |  |  |
|  | *Null model* | 20023.54 | 7803 | 16.35 | 4587.54 | -57100.03 | .50 | 5 | 7723 | all .41 (.32; .49) | all .22 (.14; .29) | all .38 (.35; .40) |  |
|  |  |  |  |  |  |  |  |  |  |  |  |  |  |
| **Language age 16** |  |  |  |  |  |  |  |  |  | - | - | - |  |
|  | *Full sex-limitation model* | 11196.67 | 4234 |  | 2728.67 | -31112.37 |  | 9 | 4243 | males .51 (.30; .65) | males .11 (.00; .29) | males .38 (.32; .45) | r_g_= .44 (.12; .50) |
|  |  |  |  |  |  |  |  |  |  | females .40 (.25; .57) | females .26 (.11; .39) | females .34 (.30; .39) | r_c_=.85 (.25; .1.0) |
|  | *Common Effects model* | 11196.79 | 4235 | 0.12 | 2726.79 | -31122.24 | .73 | 8 | 4243 | males .54 (.44; .65) | males .08 (.00; .23) | males .38 (.32; .44) |  |
|  |  |  |  |  |  |  |  |  |  | females .40 (.25; .57) | females .26 (.10; .39) | females .34 (.30; .39) |  |
|  | *Scalar Effects model* | 11199.99 | 4237 | 3.19 | 2725.99 | -31139.03 | .20 | 6 | 4243 |  |  |  |  |
|  |  |  |  |  |  |  |  |  |  |  |  |  |  |
|  | *Null model* | 11200.96 | 4238 | 0.97 | 2724.96 | -31148.05 | .32. | 5 | 4243 | all .48 (.37; .60) | all .17 (.06; .26) | all .35 (.32; .39) |  |
| **Reading Fluency age 16** |  |  |  |  |  |  |  |  |  |  |  |  |  |
|  | *Full sex-limitation model* | 10769.99 | 4010 |  | 2749.99 | -29300.69 |  | 9 | 4019 | males .69 (.52; .74) | males .00 (.00; .16) | males .31 (.26; .36) | r_g_= .50 (.23; .50) |
|  |  |  |  |  |  |  |  |  |  | females .53 (.36; .69) | females .14 (.00; .28) | females .33 (.29; .38) | r_c_=1.0 (.00; .1.0) |
|  | *Common Effects model* | 10769.99 | 4011 | 0.00 | 2747.99 | -29310.68 | 1.0 | 8 | 4019 | males .69 (.52; .74) | males .00 (.00; .16) | males .30 (.26; .36) |  |
|  |  |  |  |  |  |  |  |  |  | females .53 (.36; .69) | females .14 (.00; .28) | females .33 (.29; .38) |  |
|  | *Scalar Effects model* | 10777.15 | 4013 | 3.19 | 2751.15 | -29323.51 | .03 | 6 | 4019 |  |  |  |  |
|  |  |  |  |  |  |  |  |  |  |  |  |  |  |
|  | *Null model* | 10777.68 | 4014 | 0.53 | 2749.68 | -29332.97 | .46 | 5 | 4243 | all .64 (.52; .71) | all .04 (.00; .14) | all .32 (.29; .36) |  |
|  |  |  |  |  |  |  |  |  |  |  |  |  |  |
| **Reading-Comprehension age 16** |  |  |  |  |  |  |  |  |  |  |  |  |  |
|  | *Full sex-limitation model* | 9051.06 | 3297 |  | 2457.06 | -23894.84 |  | 9 | 3306 | males .47 (.27; .56) | males .00 (.00; .16) | males .53 (.44; .62) | r_g_= .50 (.29; .50) |
|  |  |  |  |  |  |  |  |  |  | females .52 (.46; .58) | females .00 (.00; .16) | females .48 (.42; .54) | r_c_=.97 (.00; .1.0) |
|  | *Common Effects model* | 9051.06 | 3298 | 0.00 | 2455.06 | -23904.83 | 1.0 | 8 | 3306 | males .47 (.27; .56) | males .00 (.00; .16) | males .53 (.44; .62) |  |
|  |  |  |  |  |  |  |  |  |  | females .52 (.46; .58) | females .00 (.00; .16) | females .48 (.42; .54) |  |
|  | *Scalar Effects model* | 9052.06 | 3300 | 0.99 | 2452.06 | -23923.81 | .61 | 6 | 3306 |  |  |  |  |
|  |  |  |  |  |  |  |  |  |  |  |  |  |  |
|  | *Null model* | 9052.06 | 3301 | 0.00 | 2450.06 | -23933.81 | .99 | 5 | 3306 | all .51 (.37; .56) | all .00 (.00; .11) | all .49 (.44; .55) |  |
|  |  |  |  |  |  |  |  |  |  |  |  |  |  |

Full sex limitation model estimates ace-male (3 parameters), ace-females (3 parameters), genetic (r_g_) and environmental (r_c_) correlations for DZos. Qualitative sex differences exist if r_g_ <0.50 and r_c_<1.0. r_c_ and r_g_ are estimated in two separate models: one with r_g_ freely estimated and r_c_ is constrained to be 1, and another where r_c_ is freely estimated and r_g_ is constrained to 0.50.

The nested models are fitted to test for qualitative, quantitative and variance differences in the following order:

The common effect model tests for qualitative sex differences. The model allows for quantitative sex difference by constraining r_g_=0.50 and r_c_=1.0. ace-male (3 parameters), ace-females (3 parameters) are free to be estimated separately for males and females. This way only the variance that is common to both males and females can account for differences. If this model fits significantly worse than the full model, qualitative sex differences are in place.

The scalar effect model tests for quantitative sex differences. This model further removes variance differences between males and females by constraining the ace-males to be equal to ace-females. This way only phenotypic variance across sexes is allowed. If the fit of this model is significantly worse than the common model, this indicates quantitative sex differences.

The null model tests for variance differences and because all parameters are constrained (r_g_=0.50, r_c_=1.0, ace-male= ace-females, variance-male=variance-females) it tests for the null hypothesis: that there are no sex differences. The fit comparison between the scalar and null models tests for variance differences: a worse fit of the null model indicates variance differences. If the null model is the best fit of all, then it can be concluded that there are no qualitative, quantitative or variance differences.
